# Gene regulation of adult skeletogenesis in starfish and modifications during gene network co-option

**DOI:** 10.1038/s41598-021-99521-4

**Published:** 2021-10-11

**Authors:** Atsuko Yamazaki, Shumpei Yamakawa, Yoshiaki Morino, Yasunori Sasakura, Hiroshi Wada

**Affiliations:** 1 Faculty of Life and Environmental Sciences, University of Tsukuba, Tennodai 1-1-1, Tsukuba, Ibaraki 305-8572 Japan; 2 Graduate School of Life and Environmental Sciences, University of Tsukuba, Tsukuba, Ibaraki 305-8572 Japan; 3 Shimoda Marine Research Center, University of Tsukuba, Shimoda, Shizuoka 415-0025 Japan

**Keywords:** Developmental biology, Evolutionary developmental biology

## Abstract

The larval skeleton of the echinoderm is believed to have been acquired through co-option of a pre-existing gene regulatory network (GRN); that is, the mechanism for adult skeleton formation in the echinoderm was deployed in early embryogenesis during echinoderm diversification. To explore the evolutionary changes that occurred during co-option, we examined the mechanism for adult skeletogenesis using the starfish *Patiria pectinifera*. Expression patterns of skeletogenesis-related genes (*vegf*, *vegfr*, *ets1/2*, *erg*, *alx1*, *ca1*, and *clect*) suggest that adult skeletogenic cells develop from the posterior coelom after the start of feeding. Treatment with inhibitors and gene knockout using transcription activator-like effector nucleases (TALENs) suggest that the feeding-nutrient sensing pathway activates Vegf signaling via target of rapamycin (TOR) activity, leading to the activation of skeletogenic regulatory genes in starfish. In the larval skeletogenesis of sea urchins, the homeobox gene *pmar1* activates skeletogenic regulatory genes, but in starfish, localized expression of the *pmar1*-related genes *phbA* and *phbB* was not detected during the adult skeleton formation stage. Based on these data, we provide a model for the adult skeletogenic GRN in the echinoderm and propose that the upstream regulatory system changed from the feeding-TOR-Vegf pathway to a homeobox gene-system during co-option of the skeletogenic GRN.

## Introduction

The co-option of pre-existing gene regulatory networks (GRNs) is considered to be key to the evolution of morphological novelties^1^, and together with novelties in insects, such as beetle horns^2,3^, butterfly eyespots^4^, and treehopper helmets^5^, the calcitic larval skeleton in the echinoderm is one of the best-studied experimental models of this process^6^. All five groups of extant echinoderms (echinoids [sea urchins], holothuroids [sea cucumbers], ophiuroids [brittle stars], asteroids [starfishes], and crinoids [sea lilies]) possess adult skeletons, whereas larval skeletons are formed in only echinoids, ophiuroids, and holothuroids (see Koga et al.^7^). It has been considered that the adult skeleton originally existed in the common ancestor of echinoderms, and the developmental process was recruited to early development during echinoderm diversification, leading to the innovation of the larval skeleton^8^. However, when the larval skeleton evolved is still debated; although two studies using transcriptome data indicated that it evolved independently in the echinoid and ophiuroid lineages^9,10^, a recent study that estimated the ancestral state using phylogenetic analysis based on spatial gene expression patterns proposed that larval skeletogenic cells were acquired in the common ancestor of eleutherozoans (echinoderms excluding crinoids) and that the starfish lineage lost the larval skeleton^11^.

A certain subset of GRN components is shared between larval and adult skeletogenic GRNs^12^, the former of which have been well studied using modern-type echinoids (euechinoids) (see reviews: Shasikant et al.^13^; Minokawa^14^). The paired-type homeobox gene *pmar1*/*micro1* (hereafter referred to as *pmar1*) is activated earliest in skeletogenic progenitor cells during the cleavage stage by maternal factors and activates key regulatory genes, such as *alx1*, *ets1* or *erg,* by repressing the hairy family gene *hesC*, whereas Vegf signaling independently promotes later processes, especially the behavior of the emerging skeletogenic mesenchyme cells. Previous gene expression analyses demonstrated that Vegf signaling genes and regulatory genes such as *alx1*, *ets1*, and *erg* are also associated with the formation of adult skeletons in euechinoids and starfishes^12,15,16^, but the regulatory connections among these genes during the adult skeletogenic phase are still unclear. In addition, it was suggested that no Pmar1-HesC system exists upstream of adult skeletogenic GRN^12^, and so far, the upstream regulatory system for adult skeletogenic genes has not been examined well. Thus, it is essential to reveal more details of the adult skeletogenic GRN to better understand the evolutionary modification for the innovation of the larval skeleton.

To understand the adult skeletogenic GRN in the echinoderm, we here examined the mechanism of adult skeleton formation in the starfish *Patiria pectinifera.* Based on the comparison of data from *P. pectinifera* with data from the euechinoid *Hemicentrotus pulcherrimus*, we discuss evolutionary modifications that occurred during the co-option of the skeletogenic GRN in echinoderms.

## Results

### Presumptive adult skeletogenic mesenchyme cells emerge around the posterior coelom in the starfish *P. pectinifera*

It is still unclear where adult skeletogenic cells are derived from in starfishes, including *P. pectinifera*, although later development in starfish has been well described^17,18^. Therefore, we observed adult skeleton formation until 7 days postfertilization (dpf), when skeletal rudiments were first observed on the dorsal and left sides of the stomach.

**Figure 1:**
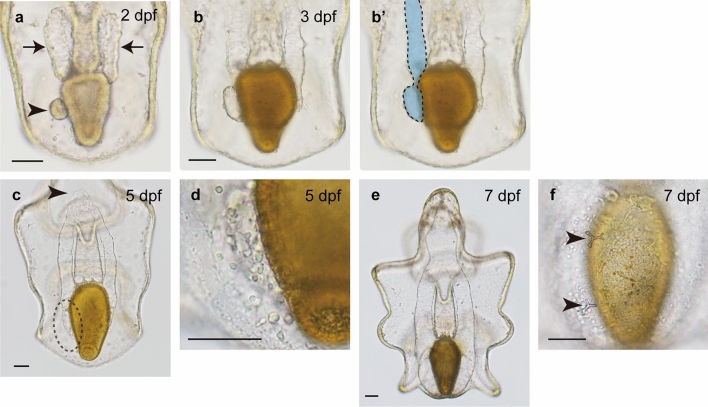
Observation of coeloms and skeletogenic mesenchyme cells in the starfish *Patiria pectinifera*. Bipinnaria larvae at 48 h postfertilization (hpf) possess two types of coeloms: a posterior enterocoel (PE, arrowhead in a) and two bilateral coelomic pouches (arrows in **a**). After the onset of feeding, the bilateral coelomic pouches extend posteriorly along the lateral walls of stomach, and the left coelomic pouch and PE fuse (**b**; the left coeloms are shown in blue encircled by dotted lines in **b’**). At 5 days postfertilization (dpf), the bilateral coeloms fuse around the pharynx (arrowhead in **c**), and a mesenchyme cell population appears in the dorsal posterior region (dotted circle in **c**; **d**, enlarged image). By 7 dpf, adult skeletal rudiments first emerge on the dorsal left side (**e**; arrowheads in **f**). Scale bars: 50 μm.

At 2 dpf, bipinnaria larvae that started feeding had two types of coelomic pouches: a posterior enterocoel (PE, arrowhead in Fig. 1a) and bilateral coelomic pouches around the pharynx (arrows in Fig. 1a). By 3 dpf, the bilateral coelomic pouches had extended posteriorly, and the left coelomic pouch was attached to the PE (Fig. 1b, b’; left coelomic pouch and PE are shown in blue with dotted lines in Fig. 1b’). By 5 dpf, the posteriorly extended left coelomic pouch have fused with PE to form the left posterior coelom, and the upper part of the bilateral coelomic pouches had fused around the mouth (arrowhead in Fig. 1c), while the mesenchyme cell population had emerged on the dorsal and left sides of larvae, i.e., near the left posterior coelom (dotted circle in Fig. 1c; enlarged image is shown in Fig. 1d). At approximately 7 dpf, the larvae had developed into brachiolaria larvae (Fig. 1e). A few adult skeletal rudiments first emerged on the dorsal and left sides of the larvae (arrowheads in Fig. 1f); skeletal fragments were also formed on the right side later.

### *Vegfr*,* ets1/2*, *erg*, and *alx1* are expressed in the posterior coelom in starfish

**Figure 2:**
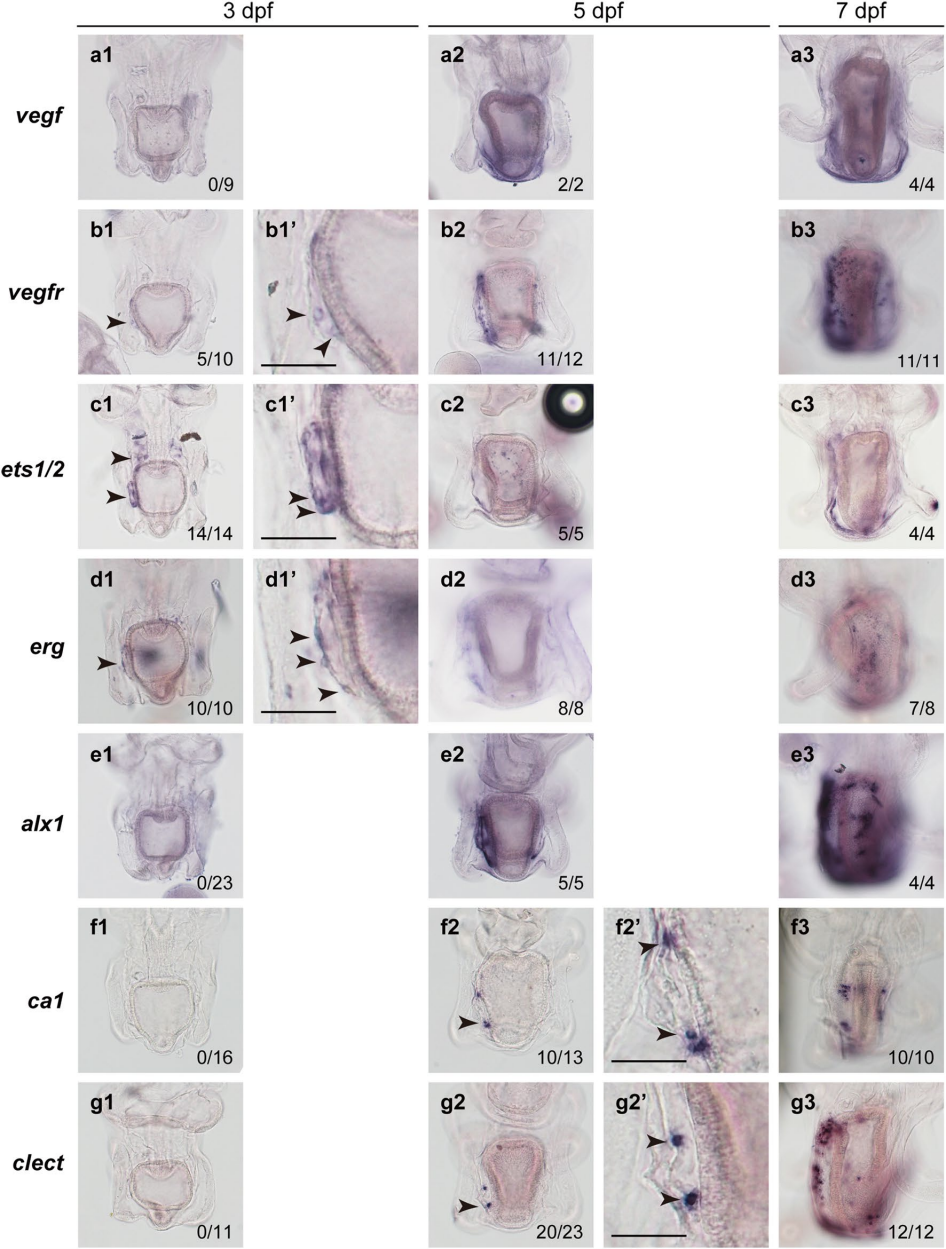
Expression of skeletogenesis-related genes in starfish larvae. Expression patterns of *vegf* (**a1**–**a3**), *vegfr* (**b1**–**b3**), *ets1/2* (**c1**–**c3**), *erg* (**d1**–**d3**), *alx1* (**e1**–**e3**), *ca1* (**f1**–**f3**), and *clect* (**g1**–**g3**) were examined at 3 dpf (**a1**–**g1**, **b1’**–**d1’**), 5 dpf (**a2**–**g2**, **f2’**, **g2’**), and 7 dpf (**a3**–**g3**) by whole-mount in situ hybridization (WMISH). The numbers shown in the lower right corner of each image indicate the number of larvae showing a positive WMISH signal among the larvae examined. Arrowheads indicate the regions or cells expressing each gene. Enlarged images are shown in (**b1’**–**d1’**, **f2’**, **g2’**). See details in the text. Scale bars: 50 μm.

We next investigated the expression of skeletogenesis-related genes (*vegf*, *vegfr*, *ets1/2* [an ortholog of sea urchin *ets1*], *erg*, *alx1*, *ca1* [*carbonic anhydrase 1*], and *clect* [*c-lectin*]) at 3, 5, and 7 dpf by whole-mount in situ hybridization (WMISH) (Fig. 2). Although we previously showed the expression of some of these genes^7,15,16^, to clarify the temporal expression profile, we reexamined gene expression patterns in a single batch because the temporal expression patterns appeared to vary slightly among batches, possibly due to differences in feeding conditions or genetic backgrounds.

At 3 dpf, expression of *vegfr*, *ets1/2*, and *erg* was detected in several cells in the left posterior coelom; the expression of *ets1/2* was also observed in the anterior coelom (Fig. 2b1–d1, enlarged image in Fig. 2b1’–d1’). WMISH signals for *vegf*, *alx1*, *ca1*, and *clect* were not detected at this stage (Fig. 2a1,e1–g1). At 5 dpf, *vegfr*, *ets1/2*, *erg*, and *alx1* expression was observed in the left posterior coelom and surrounding mesenchyme cells (Fig. 2b2–e2), while expression of *ca1* or *clect* was also detected in a few mesenchyme cells around the left posterior coelom (Fig. 2f2 and g2, enlarged image in Fig. 2f2’ and g2’). At 5 dpf and 7 dpf, the posterior ectoderm region showed the expression of *vegf* (Fig. 2a2 and a3). At 7 dpf, the expression of the remaining genes was detected in the mesenchyme cells surrounding the posterior coeloms, but the WMISH signals of *ca1* and *clect* were detected in fewer mesenchyme cells than those of *vegfr*, *ets1/2*, *erg*, or *alx1* (Fig. 2b3–g3).

### Feeding-nutrient sensing pathway regulates adult skeletogenesis in starfish

**Figure 3:**
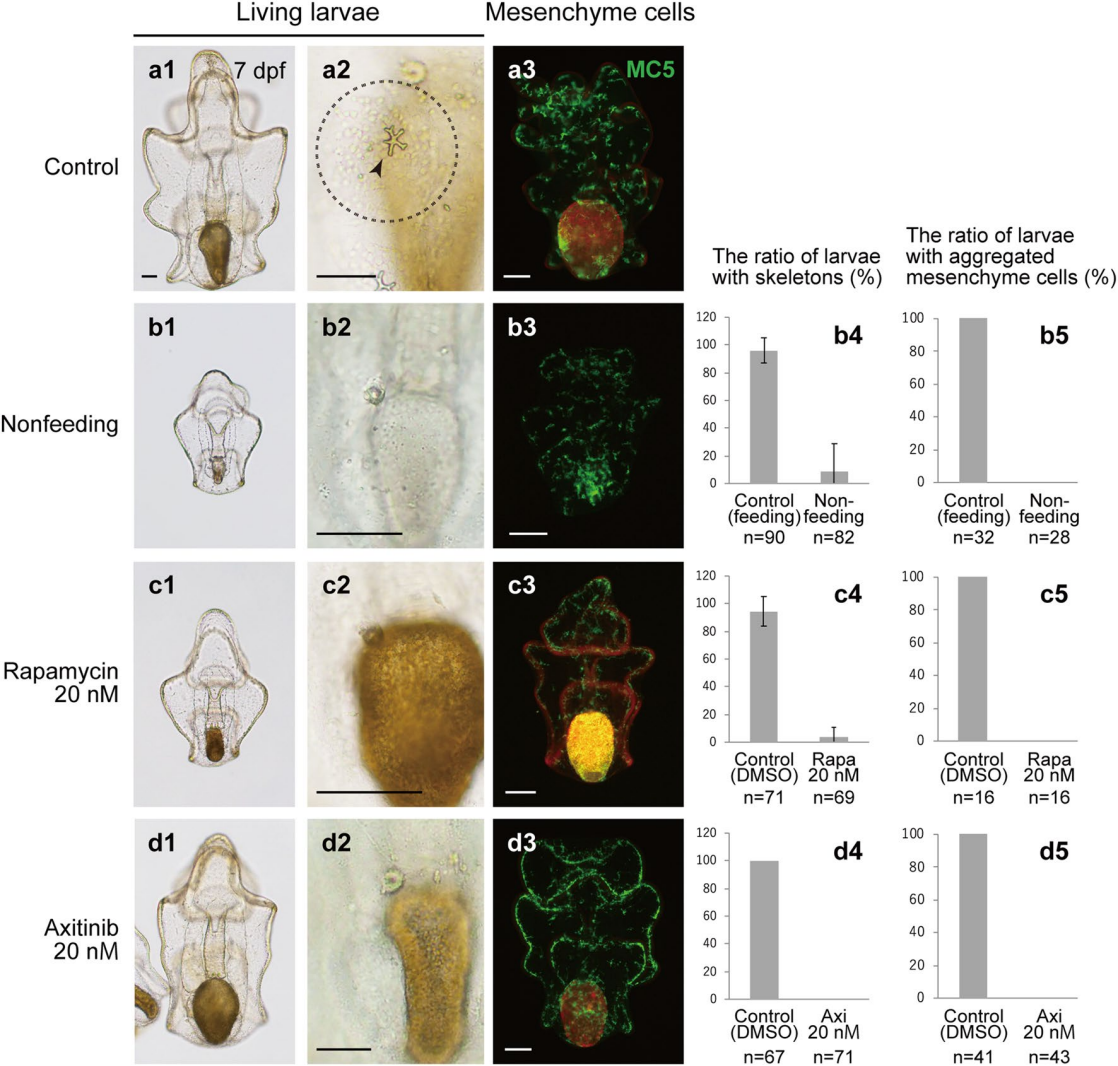
Observations of skeleton formation in the experimental starfish larvae. Morphology and skeleton formation were observed in control larvae (**a1**–**a3**), nonfeeding larvae (**b1**–**b5**), rapamycin (rapa)-treated larvae (**c1**–**c5**), and axitinib (axi)-treated larvae (**d1**–**d5**) at 7 dpf. (**a1**–**d1**, **a2**–**d2**) Living larvae. In the panel a2, the arrowhead indicates an adult skeletal rudiment and the dotted circle indicates aggregated mesenchyme cells. (**a3**–**d3**) Fluorescence images of larvae examined by immunohistochemistry using the mesenchyme-specific marker MC5. The green signal shows MC5 expression, whereas the red signal shows *Chaetoceros calcitrans* in the stomach or autofluorescence. At 7 pdf, the ratio of larvae with adult skeletons (**b4**–**d4**) and that of larvae with aggregated mesenchyme cells in the posterior dorsal region (**b5**–**d5**) were evaluated. Feeding larvae and DMSO-treated larvae were used as controls for nonfeeding larvae and inhibitor-treated larvae, respectively. Scale bars: 50 μm. M: mol/L.

The feeding-nutrient sensing pathway is considered to be an additional indispensable factor for the formation of adult rudiments in indirectly developing echinoderms. In the indirectly developing starfish, the larval stage at which adult rudiments form cannot be observed without feeding^19,20^. In addition, a previous study on sea urchins suggests that adult rudiment formation requires the activity of target of rapamycin (TOR)^21^, which has evolutionarily conserved functions in eukaryotic cell growth and metabolism with external environmental signals, including nutrients^22^. Thus, we observed skeleton formation in larvae with no feeding and those treated with an inhibitor of the TOR signaling pathway, rapamycin (Fig. 3). The starfish larvae were treated with rapamycin from 2 dpf until 7 dpf, and the treated larvae were fed from 2 dpf.

At 7 dpf, both nonfeeding and rapamycin-treated larvae showed smaller body sizes than control larvae (intact larvae with feeding and DMSO-treated larvae, respectively) (Fig. 3a1–c1). Most control starfish larvae formed skeleton rudiments in each experiment at 7 dpf (arrowheads in Fig. 3a2), but almost none of the nonfeeding or rapamycin-treated larvae formed skeletons at 7 dpf (Fig. 3b2,c2,b4,c4). Notably, among nonfeeding larvae, a small proportion of larvae formed adult skeletal rudiments, but all of the larvae with skeletons were derived from one batch (13 of 29 larvae in this batch) among the five batches examined. Aggregation of mesenchyme cells on the posterior-dorsal side was observed in the control larvae (dotted circle in Fig. 3a2) but not in the other experimental larvae (Fig. 3b2,c2,b5,c5). Nonetheless, similar to the control larvae, the nonfeeding and rapamycin-treated larvae had a certain number of mesenchyme cells expressing the mesenchyme cell marker MC5^23^ (Fig. 3a3–c3).

**Figure 4:**
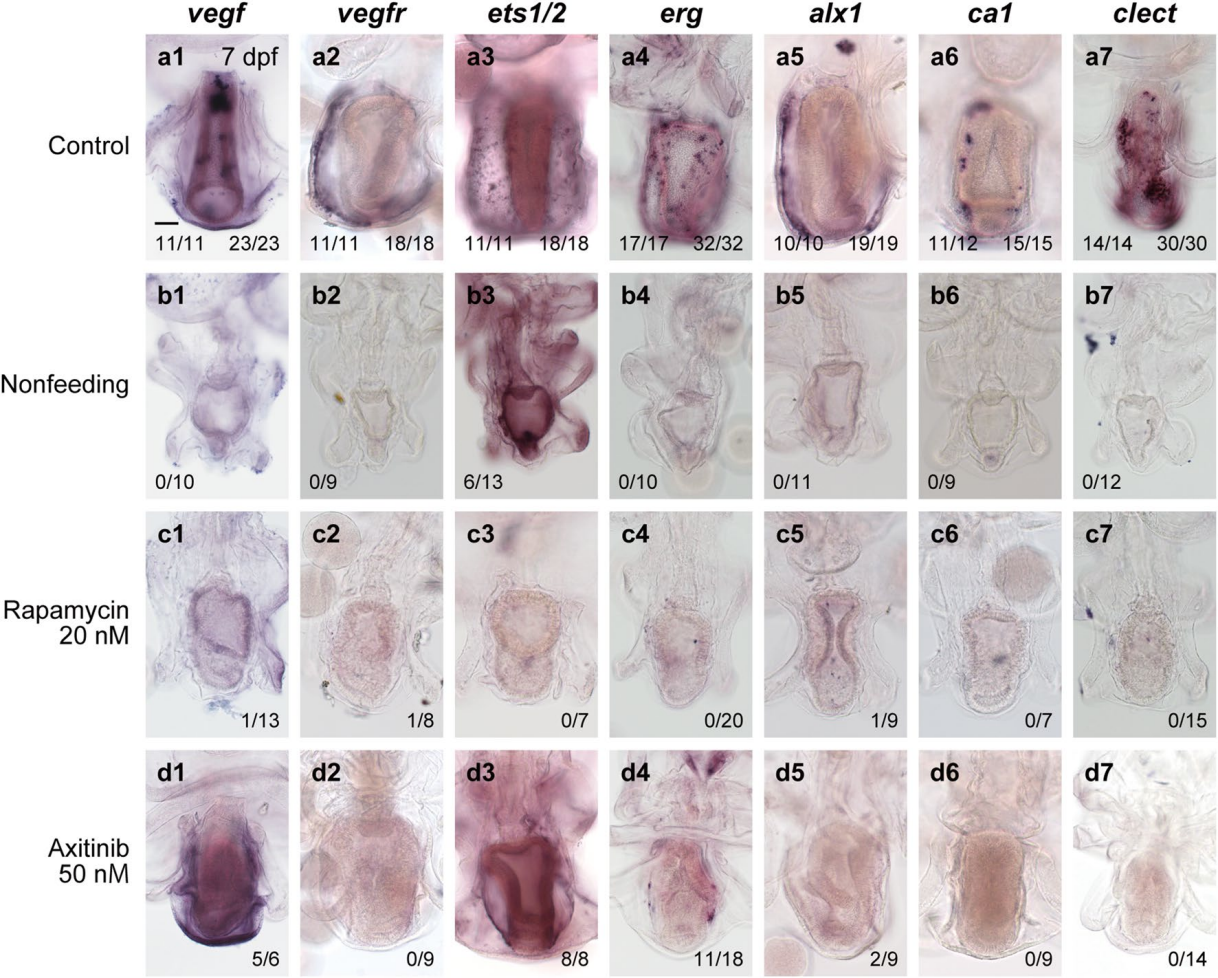
Expression of skeletogenesis-related genes in the experimental starfish larvae. The expression of *vegf* (**a1**–**d1**), *vegfr* (**a2**–**d2**), *ets1/2* (**a3**–**d3**), *erg* (**a4**–**d4**), *alx1* (**a5**–**d5**), *ca1* (**a6**–**d6**), and *clect* (**a7**–**d7**) was examined by WMISH in the control (feeding and DMSO-treated larvae) and inhibitor-treated larvae. The numbers in the lower corner of each image indicate the number of larvae showing a positive WMISH signal/the total number of larvae examined. In the control larvae, the number in the lower left corner is for feeding larvae, and that in the lower right corner is for DMSO-treated larvae. Scale bar: 50 μm. M: mol/L.

We next examined the expression patterns of *vegf*, *vegfr*, *ets1/2*, *erg*, *alx1*, *ca1* and *clect* in the nonfeeding larvae and larvae treated with rapamycin (Fig. 4). At 7 dpf, the expression of all genes other than *ets1/2* was not detected in the nonfeeding larvae (Fig. 4b1,b2,b4–b7), unlike in the control larvae (Fig. 4a1,a2,a4–a7), suggesting that feeding is essential for the expression of these genes. In contrast, *ets1/2* expression was detected in the posterior coeloms in approximately half of larvae (Fig. 4b3). In most of the rapamycin-treated larvae, no expression was detected of any of the genes examined (Fig. 4c1–c7), suggesting that TOR signaling is required for the expression of skeletogenesis-related genes, including *ets1/2*.

### Vegf signaling plays indispensable roles in adult skeletogenesis of starfish

We also examined the role of Vegf signaling in adult skeleton formation using the Vegfr inhibitor axitinib (Fig. 3d1–d5). It has been demonstrated that axitinib inhibits larval skeleton formation in sea urchins^24^. We treated starfish larvae with axitinib from 2 dpf. By 7 dpf, the axitinib-treated larvae were similar in size to the control DMSO-treated larvae (Fig. 3a1 and d1). The axitinib-treated larvae showed no mesenchyme cell aggregation or skeletal fragments in the posterior-dorsal region (Fig. 3d2,d4,d5), but MC5-positive mesenchyme cells were still observed throughout the larvae, like in the control larvae (Fig. 3a3 and d3).

The expression of *vegfr*, *ca1*, and *clect* was not detected by WMISH in any larvae treated with axitinib (Fig. 4d2,d6,d7). Some axitinib-treated larvae showed *erg* and *alx1* expression (11 of 18 larvae for *erg* and two of nine larvae for *alx1*), but the signal level in axitinib-treated larvae was much lower than that in control larvae (Fig. 4d4 and d5 compared to a4 and a5). In contrast, no obvious difference was observed in *vegf* or *ets1/2* expression between control and axitinib-treated larvae (Fig. 4d1 and d3 compared to a1 and a3). These observations suggest that Vegf signaling regulates *erg*, *alx1*, *ca1* and *clect* during the early phase of adult skeleton formation in starfish. We also suggest that Vegf signaling is required for the expression of *vegfr* itself, as demonstrated in the sea urchin embryo^25^.

To further confirm the requirement of Vegf signaling in adult skeleton formation in starfish, TALEN-mediated knockout of *vegfr* was performed. We designed TALENs targeting the Vegfr exon region encoding its tyrosine kinase domain, which is a core domain for intercellular signal transduction^26^, and injected mRNA encoding the right and left arms of the TALENs into eggs of *P. pectinifera*.

To verify the genomic cleavage of the TALEN target site, we first cloned and sequenced the genomic fragments including the target site using genomes extracted from two larvae from each experimental group at 2 dpf (see Supplementary Fig. S1 online; see Method). Although there were no deletions in the target site in five clones among the control larvae, all 18 clones showed deletions of 5–23 bases in larvae injected with the TALEN right and left arms (− 5 bp in 10 clones, − 12 bp in four clones, − 14 bp in three clones, − 23 bp in one clone; see Supplementary Fig. S1 online). Furthermore, the number of deletions was not a multiple of three in most clones (14 of 18 clones, see Supplementary Fig. S1 online), suggesting that genomic cleavage and frameshift mutations occurred in the majority of cells of the larvae injected with the TALEN right and left arms, that is, the *vegfr* knockout in *P. pectinifera* was successful.

**Figure 5:**
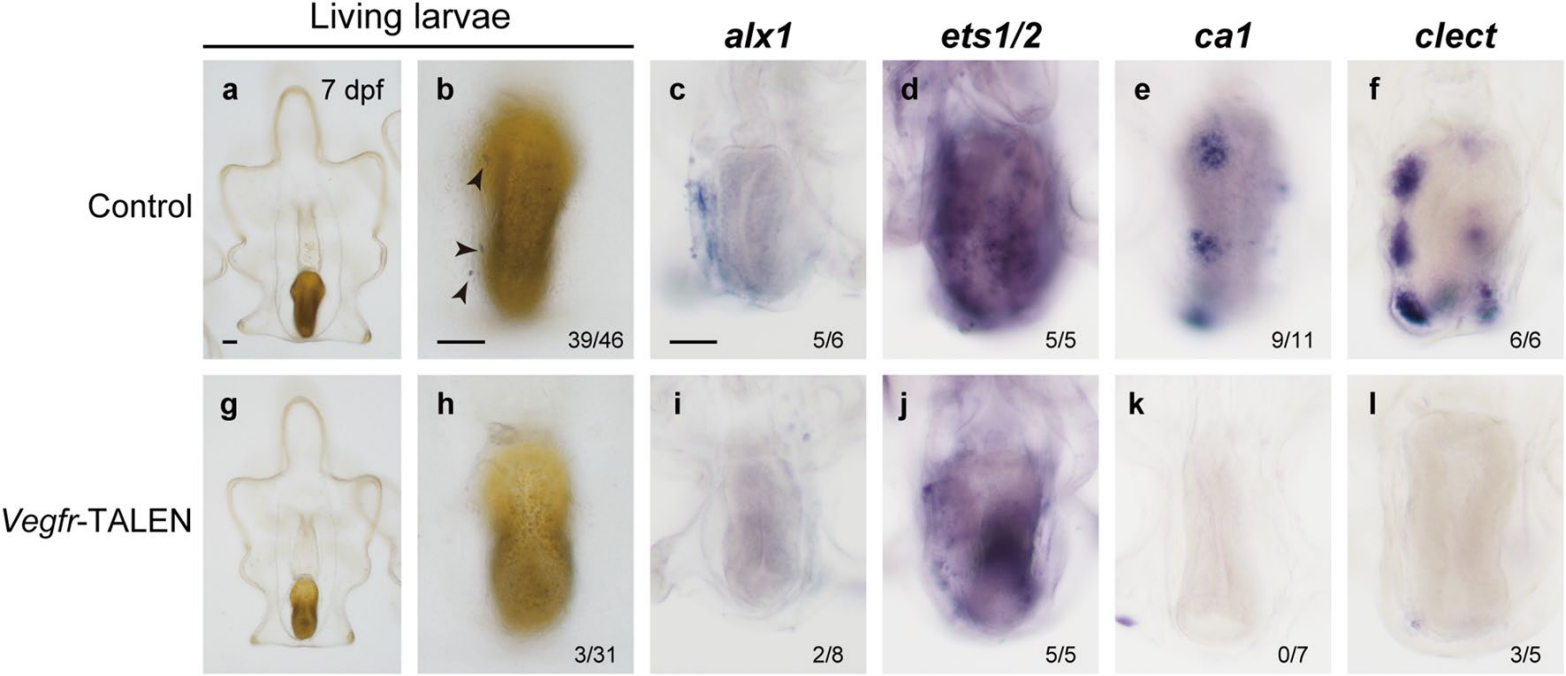
Morphology and gene expression in TALEN-mediated *vegfr* knockout starfish larvae. Adult skeleton formation and the expression of *alx1*, *ca1*, and *clect* were examined in control (**a**–**f**) and *vegfr* knockout larvae (**g**–**l**). In the panel b, arrowheads indicate adult skeletal rudiments. In panels b and h, the number in the lower right corner shows the number of larvae with skeleton rudiments/the total number of larvae examined. In the WMISH images (**c**–**f**, **i**–**l**), the number in the lower right corner shows the number of larvae with a positive WMISH signal/the total number of larvae examined. Scale bars: 50 μm.

We cultured the rest of the experimental larvae until 7 dpf. Most of the control larvae formed adult skeletons (39 of 46 larvae, Fig. 5a; arrowheads in Fig. 5b), while the proportion of larvae with skeletons was substantially lower in the *vegfr-*knockout larvae (three of 31 larvae, Fig. 5g,h). In *vegfr*-knockout larvae, mesenchyme cells were observed, as in the control larvae, but aggregation of these cells around the posterior coeloms was observed in fewer knockout larvae (three of 31 larvae) than control larvae (40 of 46 larvae). No other differences were observed in larval morphology between *vegfr*-knockout and control larvae at 7 dpf.

We investigated the expression patterns of some putative downstream genes of Vegf signaling, including *alx1*, *ets1/2*, *ca1* and *clect* (Fig. 5c–f,i–l), and found similar effects on gene expression patterns to those of axitinib treatment. Five of six control larvae showed *alx1* expression in the posterior coelom and clusters of skeletogenic mesenchyme cells (Fig. 5c), whereas two of eight *vegfr*-knockout larvae showed a positive WMISH signal of *alx1*. Note that the expression was detected in much a smaller number of cells (Fig. 5i). This incomplete suppression of *alx1* expression in *vegfr*-knockout larvae probably reflected mosaic effect of TALEN-mediated knockout. All of the control and *vegfr*-knockout larvae (five larvae each) showed *ets1/2* expression in posterior coeloms and/or mesenchyme cells, but the expression level was lower in the knockout larvae (Fig. 5d,j). *Ca1* and *clect* were also expressed in mesenchyme cell clusters around posterior coeloms in most of the control larvae (nine of 11 larvae for *ca1*; all six larvae for *clect*; Fig. 5e,f), while these expression levels were substantially lower in *vegfr*-knockout larvae (none of seven larvae for *ca1*; three of five larvae retained expression of *clect*, but in much a smaller number of cells, Fig. 5k,l). The above phenotypic effects of TALEN-mediated *vegfr* knockout were quite similar to the effects of axitinib treatment (Fig. 3). Consistent with the results of axitinib treatment, TALEN-mediated knockout experiments demonstrated that Vegf signaling regulates adult skeletogenic gene expression in starfish.

### No localized expression of the *phbA* and *phbB* genes are observed during early adult skeletogenesis in starfish

**Figure 6:**
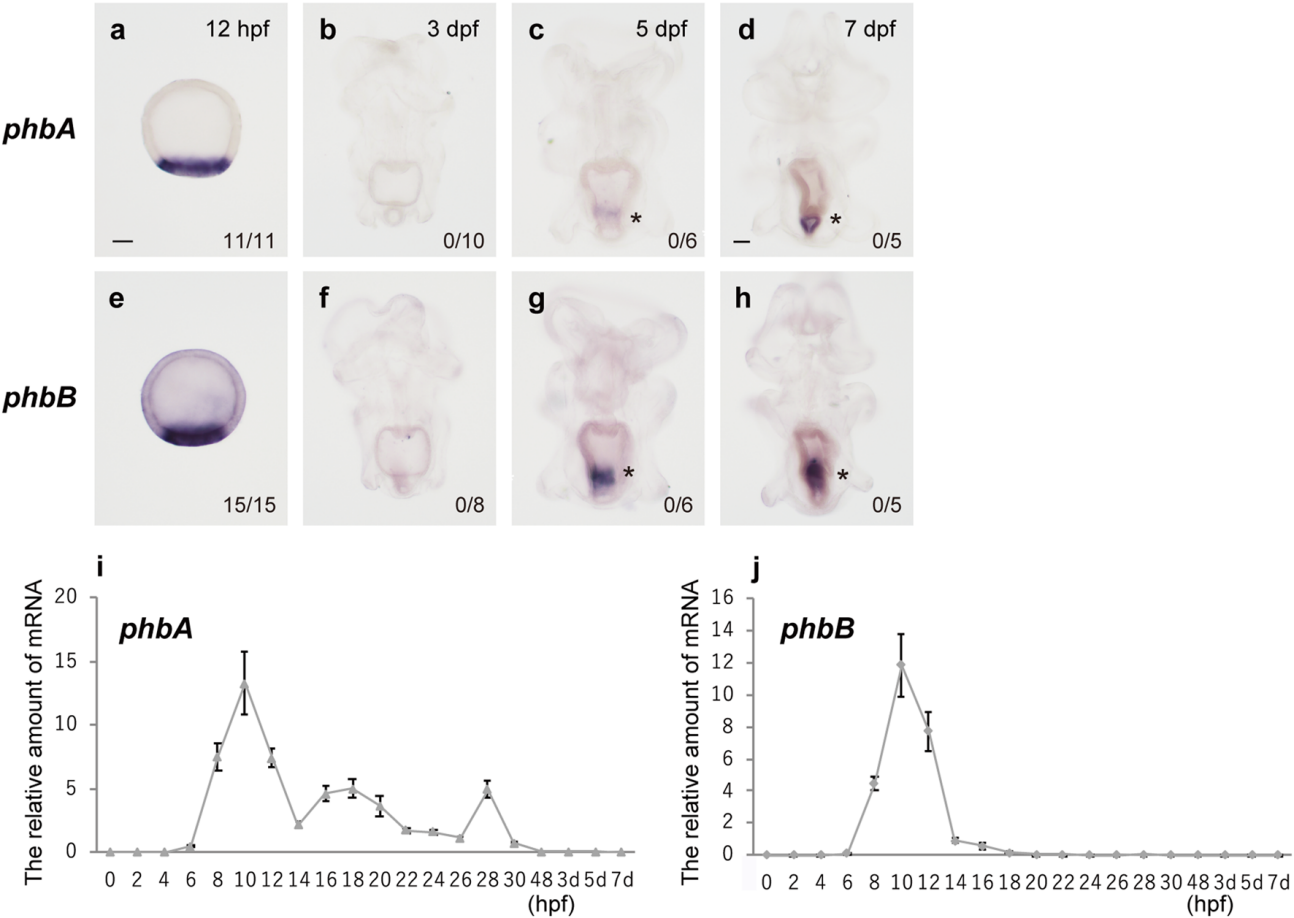
Expression of *phbA* and *phbB* in the starfish. The expression of *phbA* and *phbB* was examined by WMISH (**a**–**h**) and quantitative PCR (qPCR; **i** and **j**). (**a**–**h**) The number in the lower right corner shows the number of embryos or larvae showing a positive WMISH signal/the total number examined. Asterisks indicate nonspecific signals for *phb* genes (see “Methods”). (**i**, **j**) The X-axis shows the time after fertilization, and the Y-axis shows the mRNA amounts of *phbA* (**i**) and *phbB* (**j**) relative to that of EF1α. Data are shown as the mean ± standard deviation. Scale bars: 50 μm.

In the sea urchin larval skeletogenic GRN, Pmar1 is one of the upstream key regulators, and we previously suggested that two Pmar1-related proteins, PhbA and PhbB, function as upstream regulators in the endomesoderm specification of starfish during embryogenesis^27^. To reveal whether these two Phb proteins control adult skeleton formation, we examined the expression of *phbA* and *phbB* until 7 dpf by WMISH and quantitative PCR (qPCR) (Fig. 6).

For both *phb* genes, no obvious WMISH signals were detected at the coeloms, mesenchyme cells, or other cells from 3 to 7 dpf (Fig. 6a–h), although localized expression was detected during embryonic stages (Fig. 6a,e). We also performed qPCR to detect expression of *phb* genes. During embryonic stages, the mRNA level of both genes reached a peak at the midblastula stage (10 h postfertilization [hpf]) (Fig. 6i,j). By contrast, the expression levels of both *phb* genes were very low from 3 to 7 dpf (Fig. 6a,b); their expression levels during this period were 0.27–0.45% and 0.01–0.02% of their maximum expression levels at 10 hpf for *phbA* and *phbB*, respectively.

### Vegf and TOR signaling are not required for expression of *alx1* and *ets1* in larval skeleton formation in the sea urchin

**Figure 7:**
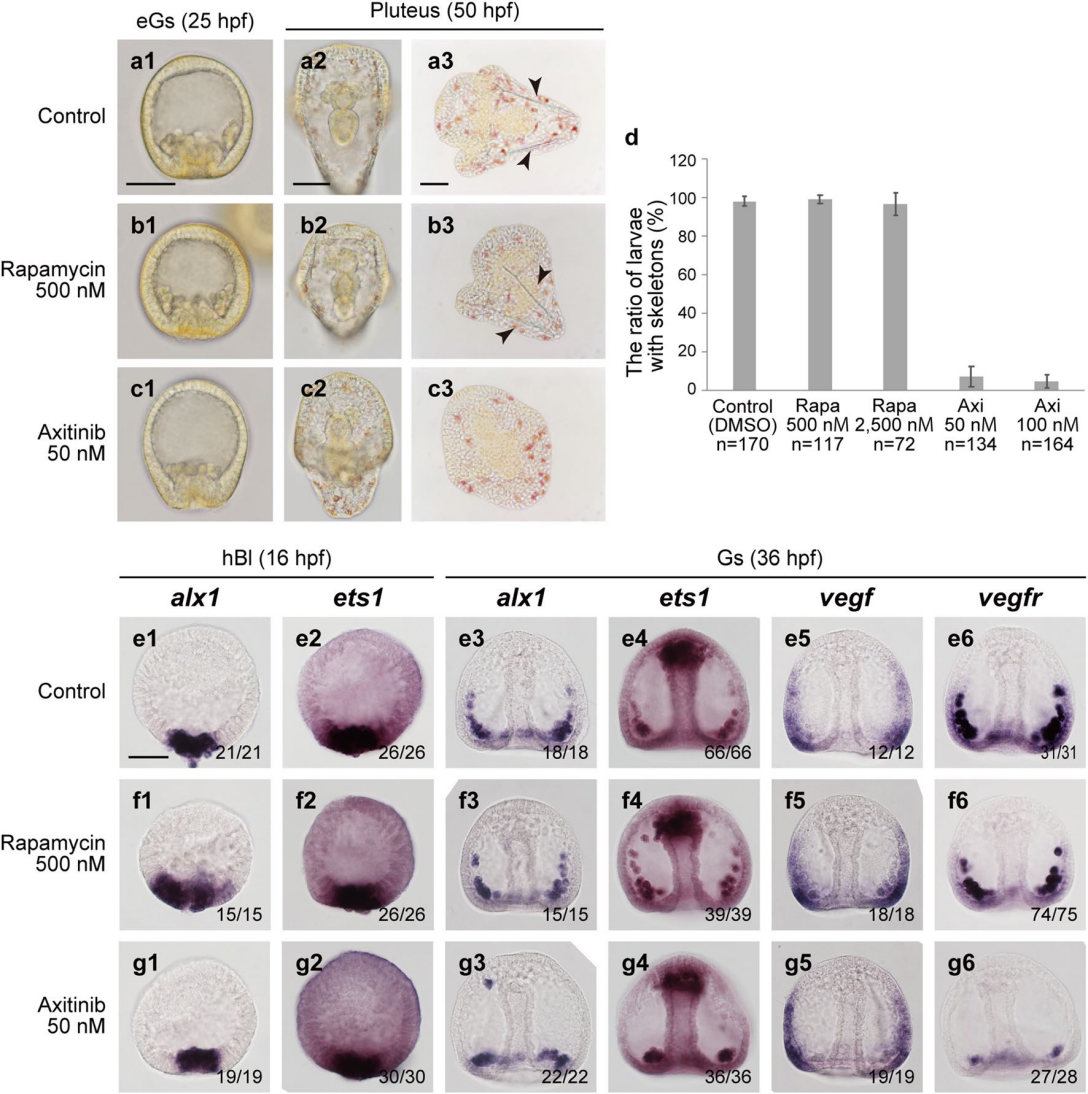
Treatment with rapamycin or axitinib in embryos of the sea urchin *Hemicentrotus pulcherrimus*. Morphology, the formation of larval skeletons, and gene expression were observed in the control (**a1**–**a3**, **e1**–**e6**), rapamycin (rapa)-treated (**b1**–**b3**, **f1**–**f6**) and axitinib (axi)-treated larvae (**c1**–**c3**, **g1**–**g6**). (**a**–**c**) Living embryo or larva at the early gastrula (eGs; 25 hpf) and pluteus (50 hpf) stages. For the observation of larval skeletons, the larvae were pressed (**a3**–**c3**). In panels of a3 and b3, arrowheads indicate larval skeletons. (**d**) The ratio of larvae with skeletons was evaluated. The total numbers of larvae examined are shown at the bottom. The expression of *alx1*, *ets1*, *vegf* and *vegfr* was examined in the control embryos treated with DMSO (**e1**–**e6**), rapamycin-treated embryos (**f1**–**f6**) and axitinib-treated embryos (**g1**–**g6**) at the hatched blastula (hBl; 16 hpf) and gastrula (Gs; 36 hpf) stages. The numbers in the lower right corner show the number of embryos showing a positive WMISH signal/the total number of embryos examined. All WMISH images except for that in panel f1 show typical expression patterns, whereas panel f1 shows an atypical expanded expression pattern (5 of 15 embryos). Scale bars: 50 μm. M: mol/L.

To estimate the evolutionary modifications in the GRN associated with co-option of skeletogenesis, we examined the effects of the Vegf and TOR signaling pathways on larval skeletogenesis in the sea urchin *H. pulcherrimus*, which belongs to the euechinoid group (Fig. 7). A previous study using another TOR inhibitor, PP242, in sea urchin embryos demonstrated that the TOR signaling pathway controls *cyclin B* mRNA translation during early cleavage stages^28^, but the role of TOR signaling in later development is still unknown. In sea urchin embryos, Vegf signaling is required for larval skeleton formation; however, this signaling pathway does not activate skeletogenic regulatory genes such as *alx1*. Zygotic expression of sea urchin *vegf* and *vegfr* starts later than the onset of *alx1* or *ets1* expression during embryonic development. *Vegfr* expression is regulated by *alx1* and *ets1* instead^25,29^.

In *H. pulcherrimus* embryos treated with rapamycin (500 nM or 2500 nM), no effects on larval skeleton formation were observed. The embryos were treated beginning at the 2-cell stage, and the cleavage of blastomeres tended to be delayed in the embryos treated with a high dose; however, primary mesenchyme cells (PMCs), which compose the skeletogenic cell population emerging before gastrulation, and larval skeletons were formed in almost all embryos (Fig. 7b1–b3,d).

We examined the expression of *alx1*, *ets1*, *vegf* and *vegfr* at the blastula and/or gastrula stages in rapamycin-treated embryos by WMISH and found that, for all the genes, the expression levels were comparable to those in the control embryos at the blastula and gastrula stages (Fig. 7e1–e6,f1–f6). A portion of rapamycin-treated blastulae (five of 15 embryos) rather showed expansion of the *alx1*-expressing region (Fig. 7f1). Thus, we obtained no evidence that TOR controls Vegf signaling or regulatory genes such as *alx1* and *ets1* during sea urchin larval skeletogenesis.

Regarding Vegf signaling, we confirmed the previous results obtained in other sea urchins^24,25^ using embryos of *H. pulcherrimus*; that is, axitinib inhibited the formation of larval skeletons (Fig. 7c2,c3,d) but not the formation of PMCs (Fig. 7c1). In the axitinib-treated embryos, expression level of *vegfr* mRNA was substantially lower than that in the control embryos (Fig. 7g6,e6), whereas the expression patterns of the other genes were not obviously affected at either stage (Fig. 7g1–g5). This result is consistent with a previous study showing that *alx1* and *ets1* expression at the gastrula stage is not downregulated in axitinib-treated embryos^30^. These observations suggest that Vegf signaling is not required for the expression of *alx1* and *ets1* at the early and later embryonic stages in the sea urchin.

## Discussion

Previous gene expression analyses of sea urchins and starfish suggested that certain larval skeletogenic GRN components identified in sea urchins, including Vegf signaling genes, are also associated with the formation of the adult skeleton in echinoderms^12,16,31^. In this study, we provide further supporting evidence and propose a model of the evolutionary change associated with the co-option of GRNs in the evolution of morphological novelties.

### Development of adult skeletogenic cells in the starfish

**Figure 8:**
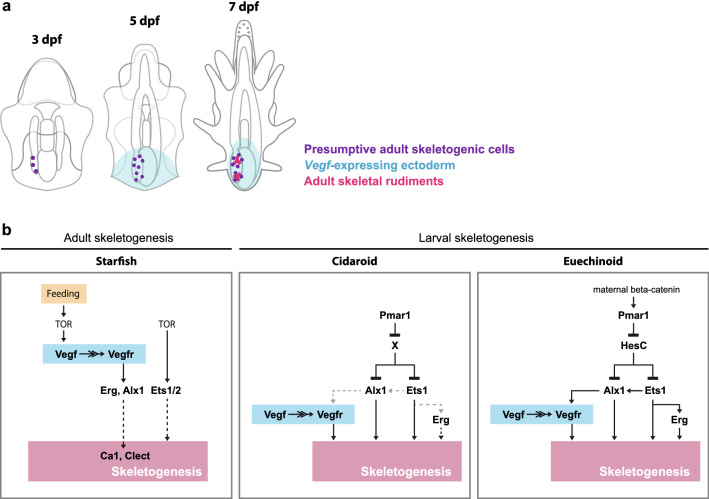
Illustration of skeletogenesis-related regions in starfish larvae (**a**) and comparison of gene regulatory network (GRN) models (**b**). (**a**) Our data suggest that adult skeletogenic cells are derived from cells included in the posterior coelom, and Vegf ligand emitted from the posterior ectoderm binds and activates the receptor Vegfr in dorsal mesenchyme cell lineage, which leads to the formation of adult skeletal rudiments on the dorsal left side of starfish larvae. (**b**) GRN models for adult skeletogenesis in starfish (left) or larval skeletogenesis in cidaroids (center) and euechinoids (right). The regulatory relationships shown by dotted lines were indicated by gene expression analyses. The cidaroid and euechinoid models are based on previous studies^27,29,45,46^.

Our observations provide insight into the cell lineage of adult skeletogenic cells in starfish. In *P. pectinifera*, a mesenchyme cell population was seen on the dorsal side of the posterior coelom by approximately 5 dpf. When the expression of skeletogenesis-related genes was observed, *vegfr*, *ets1/2* and *erg* were detected in some of the cells included in the left posterior coelom at 3 dpf, suggesting that some of the progenies develop into adult skeletogenic cells (Fig. 8a). As Yajima^32^ demonstrated that, in euechinoids, secondary mesenchyme cells (SMCs), which are a non-larval skeletogenic mesenchyme population that emerges later than PMCs during embryogenesis, contribute to adult skeletogenesis, the timing of epithelial-mesenchymal transition of adult skeletogenic cells seems to be different between starfish and sea urchins, i.e., it occurs after or before the onset of feeding, respectively.

### GRN model for adult skeletogenesis in the starfish

The hypothetical GRN model for adult skeletogenesis in starfish is shown in the left panel in Fig. 8b. Our observations suggest that adult skeleton formation in starfish requires feeding, TOR activity, and the Vegf signaling pathway, and the requirement of Vegf signaling was also confirmed by a TALEN-based knockout experiment. WMISH analysis in nonfeeding and rapamycin-treated starfish larvae suggested that the TOR-mediated feeding-nutrient sensing pathway controls adult skeletogenesis upstream of the GRN. Together with the previous studies in larvae of *C. elegans*^33^ and octopus^34^, our data suggests that the feeding-TOR pathway is conserved in bilaterians. Of note, in nonfeeding larvae, unlike in TOR-inhibited larvae, the expression of *ets1/2* in the posterior coelom was detected in approximately half of the larvae. Therefore, it seems that the expression of *ets1/2* is regulated by TOR pathway, but independently of feeding. On the other hand, inhibition of Vegf signaling caused the downregulation of genes other than *vegf* and *ets1/2*, indicating that Vegf signaling regulated by the TOR signaling pathway is required for the activation of at least some skeletogenic regulatory genes, such as *alx1* and *erg*. Since clear downregulation of *ets1/2* expression was not observed in Vegf signaling-inhibited larvae, we propose that activation of *ets1/2* is independent of Vegf signaling.

One of the notable findings in the present study is that Vegf signaling is involved in different morphogenetic processes during larval and adult skeleton formation. During larval skeletogenesis in euechinoids, *vegfr* is expressed in skeletogenic mesenchyme cells (i.e., PMCs) after their ingression into the blastocoel, and it is required for mesenchyme cells to move to the correct position and form larval skeletons^25^. In contrast, starfish larvae treated with the Vegf signaling inhibitor showed few *alx1-* or *erg*-positive cells in the posterior coelom, suggesting that specification of adult skeletogenic cells were suppressed by the Vegf signaling inhibitor. In addition, Vegf signaling differentially affects one of the key skeletogenic regulatory genes, *alx1*, in sea urchin larval skeletogenesis and starfish adult skeletogenesis; Vegf controls *alx1* expression only during adult skeletogenesis in starfish, while Vegf is dispensable for expression of sea urchin *alx1* expression in the normal development. Based on these data, we propose the following adult skeletogenic process in starfish: the TOR-mediated feeding-nutrient sensing pathway activates *vegf* in the posterior ectoderm of the larvae, and Vegf signaling from the ectoderm to the posterior coelom activates regulatory genes such as *alx1* and *erg* in the posterior coelom-mesenchyme lineage, resulting in the formation of adult skeletogenic mesenchyme cells. In contrast, *ets1/2* appears to be regulated by the TOR pathway independent of Vegf signaling (Fig. 8a; left panel in Fig. 8b).

### Evolutionary modifications in the skeletogenic GRN during co-option

We compared the adult skeletogenic pathway in the starfish with the larval skeletogenic pathway in the sea urchin to assess the evolutionary changes in GRN associated with the co-option of skeletogenesis (Fig. 8b). A double-repression system consisting of two repressors, Pmar1 and HesC, a so-called double-negative gate, activates larval skeletogenic regulatory genes in euechinoid sea urchins^35^ (Fig. 8b, right panel); this gate is not responsible for adult skeleton formation in sea urchins because *pmar1* gene expression was not detected around the adult skeleton formation site in *S. purpuratus*^12^. In this study, localized expression of the *pmar1*-related genes *phbA* and *phbB* was not detected after the onset of feeding in starfish larvae, which supports the idea that these genes do not control adult skeletogenesis. Moreover, our previous study demonstrated that a double-repression system consisting of Pmar1 and an unknown repressor other than HesC activates *alx1* and *ets1/2* in cidaroid *Prionocidaris baculosa*, which is considered to possess ancestral characteristics of sea urchins^27^ (Fig. 8b, center panel). Therefore, we propose that the upstream system was changed from the nutrient-Vegf signaling pathway to a double-negative gate with Pmar1 and an unknown repressor when the larval skeleton was acquired by co-option of the adult skeletogenic system. It should be noted that, based on the experiments using PMC-removed euechinoid embryos, the recent study also proposed that the regulation of *alx1* by Vegf signaling is ancestral mode for skeletogenic cell formation in the echinoderm^36^.

Our data suggest that the hierarchy of Vegf signaling in the skeletogenic GRN differs between larval skeletogenesis and adult skeletogenesis, occurring either upstream or downstream of *alx1*, respectively, although Vegf signaling is still required for both types of skeleton formation. A recent study on the beetle suggests that many original components are not included in the co-opted GRN^37^, and similarly, it was also suggested that a few regulatory genes, such as the t-box gene *tbr,* are included in only the larval skeletogenic GRN and not the adult GRN^12^. These facts indicate the flexibility of GRNs in nature, and future studies will be needed to understand how novel characteristics evolved in such flexible GRNs.

## Methods

### Gametes and embryos

The collection and handing of gametes of *P. pectinifera* and *H. pulcherrimus* were performed according to Koga et al.^15^ Embryos of these species were cultured in artificial seawater (MARINE ART BR, Osaka Yakken Co., Ltd.) at 22 °C and 14 °C, respectively. Starfish larvae were cultured according to the method described previously^38^ and fed *Chaetoceros calcitrans* after 2 dpf.

### WMISH and immuno-staining

The fixation, hybridization and staining of larvae or embryos were performed as described previously^27^. The sequence of *P. pectinifera erg* (*Pp-erg*) was obtained using PCR with primers that corresponds the *erg* sequence in another starfish *P. miniata*, and the orthology was confirmed by phylogenetic analysis using RAxML 8.2.12^39^ (see Supplementary Fig. S2 online). The *P. pectinifera clect* gene (*Pp-clect*) was obtained from the transcriptome assembly^38^ by BLAST search using *Sp-C-lectin* (Echinobase ID: SPU_027906) for query. The nucleotide sequences of *Pp-clect* and *Pp-erg* are shown in Supplementary text. The primers used for amplification of cDNA fragments for RNA probes were as follows: Pp-erg-F, 5′-AGATCATCAGGATGAAGCAGGAG-3′; Pp-erg-R, 5′-TCAGTTTCACGATTAAAAATAACCACA-3′; Pp-clect-F, 5′-GCACACGAGTTCGCGATGCTGTAGACTAGG-3′; Pp-clect-R-T3, 5′-ATTAACCCTCACTAAAGGGAAAAATCCCGTTGCCAACATT-3′; BamHI-Hp-vegf-F, 5′-GGGGATCCTTCAAACGCGTCGTGGTCGT-3′; BamHI-Hp-vegf-R, 5′-GGGGATCCTATCATCTCAGAAACCGAGA-3′; Hp-vegfr-F, 5′-TGTTGTTGTTGCTCTCATTATTGTT-3′; and Hp-vegfr-R, 5′-TTCCATTCAATGTCATTACTCTGTG-3′. RNA probes for the other genes were prepared as described previously^15,16,27^. The MC5 antibody was used for the visualization of mesenchyme cells in the starfish larvae; fixation and staining were performed according to the method of Hamanaka et al.^23^ In the larvae examined using RNA probes of both *phb* genes, a WMISH signal was detected in the intestine of larvae (Fig. 6c,d,g,h), but we do not consider this signal to be *phb*-specific, as mentioned previously^38^, because it is frequently detected with other RNA probes in this species.

### QPCR

qPCR analysis was performed as described previously^27^. The sequences of the primers used were as follows: Pp-phbA-qF, 5′-ACGGCAGAGCAGAGACATCA-3′; Pp-phbA-qR, 5′-TTCTGGAACCAAACCTGAACC-3′; Pp-phbB-qF, 5′-ATCGGCTTCTCCACCCAGT-3′; Pp-phbB-qR, 5′-GGAGTGCTGGAGGATGTGTG-3′; Pp-EF1α-qF, 5′-GCGTGAGCGAGGTATCACAAT-3′; and Pp-EF1α-qR, 5′-ACAATCAGCACCGCACAATC-3′.

### Treatment with inhibitors

Two inhibitors, rapamycin (AdipoGen Life Sciences) and axitinib (Selleck Chemicals LLC), were used to inhibit the TOR and Vegf signaling pathways, respectively. For the starfish *P. pectinifera*, larvae were treated with inhibitors (20 nM for rapamycin and 20 or 50 nM for axitinib) from the onset of feeding (i.e., 2 dpf) until 7 dpf. For the sea urchin *H. pulcherrimus*, embryos were treated with rapamycin (500 or 2500 nM), or axitinib (500 or 100 nM) from the 2-cell stage. Based on the trials with a series of concentrations referring to previous studies^21,24^, we chose the highest concentration that did not result in general abnormal development.

### TALEN-mediated gene knockout

The sequence of *vegfr* was obtained from the transcriptome data of the starfish *P. pectinifera*^38^. We selected tyrosine kinase domain of *vegfr* as the target of TALENs, and the location of the domain was predicted by the NCBI Conserved Domain Search^40^ (https://www.ncbi.nlm.nih.gov/Structure/cdd/wrpsb.cgi). The target sites of TALENs were finally determined using TAL Effector Nucleotide Targeter 2.0 (https://tale-nt.cac.cornell.edu/node/add/talen). The designed TALEN pairs were constructed onto the TALEN backbone vector^41^ by the Golden Gate method^42^ using the Planinum TALEN kit^43^. We used the mMESSAGE mMACHINE T3 Transcription Kit (Invitrogen) for mRNA transcription.

TALEN mRNA was introduced into eggs by microinjection according to the method of Saito et al.^44^ We injected 1000 ng/µl mRNA each of the TALEN right and left arms into the unfertilized eggs of *P. pectinifera* to make *vegfr*-knockout embryos, and 2000 ng/µl mRNA of the right-arm TALEN was used as a control. To verify the cleavages of the target site, we extracted the genomes of two 2-dpf larvae from each experimental group using Nucleo Spin Tissue (MACHEREY–NAGEL), and amplified genomic fragments including the target site by PCR. We then cloned these fragments with pGEM-T Easy Vector System (Promega), and sequenced 5 and 18 clones from the genomes of control and *vegfr*-TALEN larvae, respectively. The sequences of the primers used were as follows: F1, 5′-ACCTGCCATATGATCCTAAGTGGGAGTTCC-3′; F2, 5′-GACAAGTCACCGTGTTCATATTCACACTCA-3′; R1, 5′-GAGTTCAGTCATCAGTGCTTTCCTCTCCAC-3′ and R2, 5′-TGAACCTACTGCGTCCTTCTAGTGAAGCTG-3′. In addition, we confirmed mCherry fluorescence at approximately 24 hpf, suggesting that the TALEN mRNAs were correctly translated. Larvae were cultured as described above until 7 dpf.

## Supplementary Information

Supplementary Information.

## Data Availability

The datasets supporting the conclusions of this article are included within the article and its Supplementary information file, and available on reasonable request. The sequences for *Pp-erg* and *Pp-clect* genes are shown in the Supplementary data online.

## References

[1] Monteiro A (2012). Gene regulatory networks reused to build novel traits: Co-option of an eye-related gene regulatory network in eye-like organs and red wing patches on insect wings is suggested by optix expression. BioEssays.

[2] Moczek AP, Nagy LM (2005). Diverse developmental mechanisms contribute to different levels of diversity in horned beetles. Evol. Dev..

[3] Moczek AP, Rose DJ (2009). Differential recruitment of limb patterning genes during development and diversification of beetle horns. Proc. Natl. Acad. Sci. USA.

[4] Monteiro A (2015). Origin, development, and evolution of butterfly eyespots. Annu. Rev. Entomol..

[5] Fisher CR, Wegrzyn JL, Jockusch EL (2020). Co-option of wing-patterning genes underlies the evolution of the treehopper helmet. Nat. Ecol. Evol..

[6] Hinman VF, Cheatle Jarvela AM (2014). Developmental gene regulatory network evolution: Insights from comparative studies in echinoderms. Genesis.

[7] Koga H (2016). Experimental approach reveals the role of alx1 in the evolution of the echinoderm larval skeleton. PLoS ONE.

[8] Khor JM, Ettensohn CA (2017). Functional divergence of paralogous transcription factors supported the evolution of biomineralization in echinoderms. Elife.

[9] Telford MJ (2014). Phylogenomic analysis of echinoderm class relationships supports Asterozoa. Proc. Biol. Sci..

[10] Dylus DV, Czarkwiani A, Blowes LM, Elphick MR, Oliveri P (2018). Developmental transcriptomics of the brittle star Amphiura *filiformis* reveals gene regulatory network rewiring in echinoderm larval skeleton evolution. Genome Biol..

[11] Erkenbrack EM, Thompson JR (2019). Cell type phylogenetics informs the evolutionary origin of echinoderm larval skeletogenic cell identity. Commun. Biol..

[12] Gao F, Davidson EH (2008). Transfer of a large gene regulatory apparatus to a new developmental address in echinoid evolution. Proc. Natl. Acad. Sci. USA.

[13] Shashikant T, Khor JM, Ettensohn CA (2018). From genome to anatomy: The architecture and evolution of the skeletogenic gene regulatory network of sea urchins and other echinoderms. Genesis.

[14] Minokawa T (2017). Comparative studies on the skeletogenic mesenchyme of echinoids. Dev. Biol..

[15] Koga H (2010). Functional evolution of Ets in echinoderms with focus on the evolution of echinoderm larval skeletons. Dev. Genes Evol..

[16] Morino Y (2012). Heterochronic activation of VEGF signaling and the evolution of the skeleton in echinoderm pluteus larvae. Evol. Dev..

[17] Hyman LH (1955). The Invertebrates: Echinodermata, the Coelomate Bilateria.

[18] Irie Y, Shirai H (1998). Metamorphosis of *Patiria pectinifera*-assemblage of hydrolobe and adult rudiment: (in Japanese). Contribut. Ushimado Mar. Lab. Okayama Univ..

[19] Pace DA, Manahan DT (2007). Efficiencies and costs of larval growth in different food environments (Asteroidea: Asterina miniata). J. Exp. Mar. Biol. Ecol..

[20] Caballes CF, Pratchett MS, Kerr AM, Rivera-Posada JA (2016). The role of maternal nutrition on oocyte size and wuality, with respect to early larval development in the coral-eating starfish, *Acanthaster planci*. PLoS ONE.

[21] Carrier TJ, King BL, Coffman JA (2015). Gene expression changes associated with the developmental plasticity of sea urchin larvae in response to food availability. Biol. Bull..

[22] Saxton RA, Sabatini DM (2017). mTOR signaling in growth, metabolism, and disease. Cell.

[23] Hamanaka G (2011). Uneven distribution pattern and increasing numbers of mesenchyme cells during development in the starfish, *Asterina pectinifera*. Dev. Growth Differ..

[24] Adomako-Ankomah A, Ettensohn CA (2013). Growth factor-mediated mesodermal cell guidance and skeletogenesis during sea urchin gastrulation. Development.

[25] Duloquin L, Lhomond G, Gache C (2007). Localized VEGF signaling from ectoderm to mesenchyme cells controls morphogenesis of the sea urchin embryo skeleton. Development.

[26] Koch S, Claesson-Welsh L (2012). Signal transduction by vascular endothelial growth factor receptors. Cold Spring Harb. Perspect. Med..

[27] Yamazaki A (2020). pmar1/phb homeobox genes and the evolution of the double-negative gate for endomesoderm specification in echinoderms. Development.

[28] Chassé H (2016). Cyclin B translation depends on mTOR activity after fertilization in sea urchin embryos. PLoS ONE.

[29] Oliveri P, Tu Q, Davidson EH (2008). Global regulatory logic for specification of an embryonic cell lineage. Proc. Natl. Acad. Sci. USA.

[30] Morgulis M (2019). Possible cooption of a VEGF-driven tubulogenesis program for biomineralization in echinoderms. Proc. Natl. Acad. Sci. USA.

[31] Koga H, Morino Y, Wada H (2014). The echinoderm larval skeleton as a possible model system for experimental evolutionary biology. Genesis.

[32] Yajima M (2007). A switch in the cellular basis of skeletogenesis in late-stage sea urchin larvae. Dev. Biol..

[33] O'Donnell MP, Chao PH, Kammenga JE, Sengupta P (2018). Rictor/TORC2 mediates gut-to-brain signaling in the regulation of phenotypic plasticity in *C. elegans*. PLoS Genet..

[34] de la Serrana DG (2020). Regulation of growth-related genes by nutrition in paralarvae of the common octopus (*Octopus vulgaris*). Gene.

[35] Revilla-i-Domingo R, Oliveri P, Davidson EH (2007). A missing link in the sea urchin embryo gene regulatory network: hesC and the double-negative specification of micromeres. Proc. Natl. Acad. Sci. USA.

[36] Ettensohn CA, Adomako-Ankomah A (2019). The evolution of a new cell type was associated with competition for a signaling ligand. PLoS Biol..

[37] Hu Y (2018). A morphological novelty evolved by co-option of a reduced gene regulatory network and gene recruitment in a beetle. Proc. Biol. Sci..

[38] Yamakawa S, Morino Y, Honda M, Wada H (2018). The role of retinoic acid signaling in starfish metamorphosis. EvoDevo.

[39] Stamatakis A (2014). RAxML version 8: A tool for phylogenetic analysis and post-analysis of large phylogenies. Bioinformatics.

[40] Lu S (2020). CDD/SPARCLE: The conserved domain database in 2020. Nucleic Acids Res..

[41] Yoshida K (2017). Germ cell regeneration-mediated, enhanced mutagenesis in the ascidian *Ciona intestinalis* reveals flexible germ cell formation from different somatic cells. Dev. Biol..

[42] Cermak T (2011). Efficient design and assembly of custom TALEN and other TAL effector-based constructs for DNA targeting. Nucleic Acids Res..

[43] Sakuma T (2013). Repeating pattern of non-RVD variations in DNA-binding modules enhances TALEN activity. Sci. Rep..

[44] Saito S (2017). Characterization of TRPA channels in the starfish *Patiria pectinifera*: Involvement of thermally activated TRPA1 in thermotaxis in marine planktonic larvae. Sci. Rep..

[45] Erkenbrack EM, Davidson EH (2015). Evolutionary rewiring of gene regulatory network linkages at divergence of the echinoid subclasses. Proc. Natl. Acad. Sci. USA.

[46] Erkenbrack EM, Petsios E (2017). A conserved role for VEGF signaling in specification of homologous mesenchymal cell types positioned at spatially distinct developmental addresses in early development of sea urchins. J. Exp. Zool. B.

