# Supplementary material for: Gene regulation of adult skeletogenesis in starfish and modifications during gene network co-option
Source: Sci Rep. 2021 Oct 11;11:20111. doi: 10.1038/s41598-021-99521-4 (PMC8505446; doi:10.1038/s41598-021-99521-4)
Supplement: Supplementary file 1 — Supplementary Information. [file 41598_2021_99521_MOESM1_ESM.pdf]

Supplementary information

Gene regulation of adult skeletogenesis in starfish and modifications during gene network co-option

Authors: Atsuko Yamazaki[1*], Shumpei Yamakawa[2*], Yoshiaki Morino[1], Yasunori Sasakura[3], Hiroshi Wada[1]

[*]Equal contribution

Affiliations:

[1]Faculty of Life and Environmental Sciences, University of Tsukuba, Tsukuba, Ibaraki 305-8572, Japan

[2]Graduate School of Life and Environmental Sciences, University of Tsukuba, Tsukuba, Ibaraki, 305-8572, Japan

[3]Shimoda Marine Research Center, University of Tsukuba, Shimoda, Shizuoka, 415-0025, Japan

Corresponding authors:

Atsuko Yamazaki, Faculty of Life and Environmental Sciences, University of Tsukuba, Tennodai 1-1-1, Tsukuba, Ibaraki 305-8572, Japan. Email: yama0205@gmail.com

Hiroshi Wada, Faculty of Life and Environmental Sciences, University of Tsukuba, Tennodai 1-1-1, Tsukuba, Ibaraki 305-8572, Japan. Email: hwada@biol.tsukuba.ac.jp

Supplementary information

Figure S1. Target sequences from larvae injected with transcription activator-like effector nuclease (TALEN) mRNAs.

	Left arm / Right arm	
Target	TCCTTGGGCAGGGGGCATTCGGCAGGGTAGTCAAAGCAGCTGCCTTTGGCA	
Control	TCCTTGGGCAGGGAGCATTCGGCAGGGTAGTCAAGGCAGCTGCCTTTGGCA	0 bp
(n=5)	TCCTTGGGCAGGGGGCATTCGGCAGGGTAGTCAAAGCAGCTGCCTTTGGCA	(5/5)
	TCCTTGGGCAGGGGGCATTCGGCAGGGTAGTCAAAGCAGCTGCCTTTGGCA	
	TCCTTGGGCAGGGAGCATTCGGCAGGGTAGTCAAGGCAGCTGCCTTTGGCA	
	TCCTTGGGCAGGGAGCATTCGGCAGGGTAGTCAAGGCAGCTGCCTTTGGCA	
TALEN	TCCTTGGGCAGGGAGCATTCGGCAG-----TCAAAGCAGCTGCCTTTGGCA	-5 bp
(n=18)	TCCTTGGGCAGGGAGCATTCGGCAG-----TCAAAGCAGCTGCCTTTGGCA	(10/18)
	TCCTTGGGCAGGGAGCATTCGGCAG-----TCAAAGCAGCTGCCTTTGGCA	
	TCCTTGGGCAGGGAGCATTCGGCAG-----TCAAAGCAGCTGCCTTTGGCA	
	TCCTTGGGCAGGGAGCATTCGGCAG-----TCAAAGCAGCTGCCTTTGGCA	
	TCCTTGGGCAGGGAGCATTCGGCAG-----TCAAAGCAGCTGCCTTTGGCA	
	TCCTTGGGCAGGGAGCATTCGGCAG-----TCAAAGCAGCTGCCTTTGGCA	
	TCCTTGGGCAGGGAGCATTCGGCAG-----TCAAAGCAGCTGCCTTCGGCA	
	TCCTTGGGCAGGGAGCATTCGGCAG-----TCAAAGCAGCTGCCTTTGGCA	
	TCCTTGGGCAGGGAGCATTCGGCAG-----TCAAAGCAGCTGCCTTTGGCA	
	TCCTTGGGCAGGGAGCATTCG--------------GCAGCTGCCTTTGGCA	-14 bp
	TCCTTGGGCAGGGAGCATTCG--------------GCAGCTGCCTTTGGCA	(3/18)
	TCCTTGGGCAGGGAGCATTCG--------------GCAGCTGCCTTTGGCA	
	TCCTTGGGCAGGGAGCATTC------------AAAGCAGCTGCCTTTGGCA	-12 bp
	TCCTTGGGCAGGGAGCATTC------------AAAGCAGCTGCCTTTGGCA	(3/18)
	TCCTTGGGCAGGGAGCATTC------------AAAGCAGCTGCCTTTGGCA	
	TCCTTGGGCAGGGAGCAT-----------------------GCCTTTGGCA	-23 bp
	TCCTTGGGCAGGGAGCA------------GTCAAAGCAGCTGCCTTTGGCA	-12 bp

Each sequence shows the target sequence of our designed TALEN and is from the control or TALEN mRNA-injected embryos. Target sequences included an 18-bp left-arm binding site, a 16-bp spacer region and a 17-bp right-arm binding site (the left and right binding sites are highlighted in orange). No insertions or deletions were found in genomes from control embryos (0 bp, 5/5), while various deletions were detected in genomes from TALEN mRNA-injected embryos (-5 bp, 10/18; -14 bp, 3/18; -12 bp, 4/18; and -23 bp, 1/18). Such deletions were detected in left/right arm binding sites and spacer regions. Single base mismatch was found at the left binding site of the clone from the control embryos (indicated by the orange letter “A” in the top sequence). This substitution was considered an endogenous polymorphism of the starfish.

Figure S2. Molecular phylogenetic tree of Ets-family genes for identification of *erg* gene.

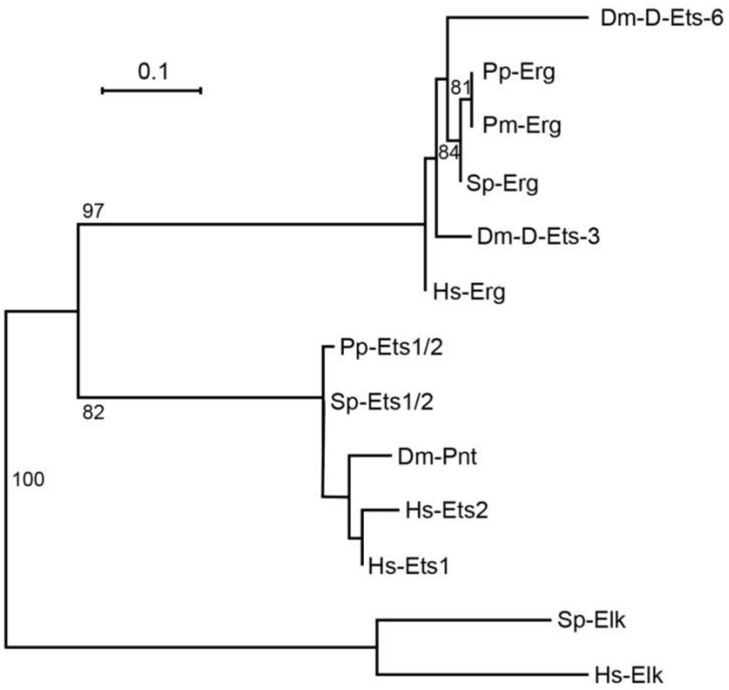

The numbers at the nodes are the bootstrap values (only those ≥ 50% are shown), obtained by 1000 replicates. Tree was constructed based on the amino acid sequences of the Ets domain using the maximum likelihood (ML) method. The amino acid substitution model was LG + G + F. Accession numbers are as follows: Dm-D-Ets-6, NP_001285547.1; Pm-erg, ADA79646.1; Sp-erg, NP_999833.1; Dm-D-Ets-3, NP_729142.1; Hs-Erg, NP_001317954.1; Pp-Ets1/2, BAJ33504.1; Sp-Ets1/2, NP_999698.1; Dm-Pnt, NP_524461.2; Hs-Ets2, NP_001243224.1; Hs-Ets1, NP_001155894.1; Sp-Elk, NP_999792.1; Hs-Elk, AAH48296.1. Abbreviations: Hs, *Homo sapiens*; Dm, *Drosophila melanogaster*; Sp, *Strongylocentrotus purpuratus*; Pp, *Patiria pectinifera*; Pm, *Patiria miniata*.

Supplementary data

The Sequences for *Pp-erg and Pp-clect*

Underlines indicate for sequences that correspond to primers. The sequences of *Pp-erg* and *Pp-clect* were obtained from PCR amplification using cDNA and transcriptomic data, respectively.

>Pp-erg

AGATCATCAGGATGAAGCAGGAGCCGGACCACGGAAGTCACGGCCACGAGGCCGGTAGCCTCGGCG
GGCGGGTGGTGGAAGCCGAGTCCCCGTTGGACTGCAGCGTGACCAAGAGGCAACCCCACGGGGCTAT
GACGGAGGCCACCCAGGCGGCACCCCCTTACCCGTGCCCGCCTTACCCCGGATCGGAATCGGACCGTG
CTCGCTCACCCCCGCCCAATGTCACAACCAACGAGAAACGGGTCATTGTGCCTGCTGACCCCAACATGT
GGACGATCGACCACGTGCAGCAGTGGGTACAGTGGGCGGTCAGGGAGTACTCCCTGCAGGACGTGCT
GGTCAGTCGGTTCGGCATTGACGGCAAACAGCTCTGCAAGATGACCCGCGAGGACTTCACGCGACTGA
CCAGCTCCTACAACGCCGACGTGCTCCTCTCCCATCTCAACTTCCTCAAGCAAGCCCCACTGCCAAACCT
GACATCAGACGACGTAGACAAAGCTCTTCAGCCTTCCCCCAGGGCTCCGCCTAGCTCACAAGCAACGAA
CATTGGAACGGCTCAGCCCATCACCGAGAAGAAATACACGTGCAATGCTACAAATTACTACCCGGATCA
GATGCAAAAAGCAAGTGCCAGTGCGTTCCCGTACCCAGTCAGCTCCCACGTGGACACAAATTCCCGGA
TGCAACGAACAGAGCCTTCGTGCGCTGAGTCGCTGGTGCGCGGCAGGCAAAACAGCTGGGCCTCGTCT
GTGCCTGTGTCCTCCTCAAAAGGATTTACACAGACGACGACGTCAATTCCCAAGGCTGGCATCGAATCC
TCATCCCAGATCAGACCAGATCCGTATCAAGTCTTCGGTCCCACCAGCAGAACGCTTGCAAATCCAGAA
AACCCAGCAAGCAGCTTAGGAGGACATGTCTGGAACCGGAGTGGTCAGATCCAGCTGTGGCAGTTCCT
ACTGGAGCTCCTGTCCGACAGCTCCAACGCCAACTGCATCACGTGGGAGGGCACCAACGGGGAGTTCA
AGATGACCGACCCGGACGAGGTGGCCCGCCGCTGGGGCGAGCGCAAGAGCAAACCCAACATGAACTA
CGACAAGCTCAGCCGGGCCCTGCGCTACTACTACGACAAGAACATTATGACCAAGGTCCACGGCAAGC
GCTACGCCTACAAGTTCGACTTCGCGGGGCTGGCCCAGGCCATGCAGCCGGTGCAGGCCGACCCCAGC
GTCTACCGTTACCAGTCGGACTTGAGCTACCTGGCCCAGGGCTATCACCACCCCAGCAAGATCAACTTT
GTGGGCACGCCCATCCCCTCCACCAACGCCGGCCTCTTCAGCTCGCACGGATCCTATTGGTCGTCGCCC
AGCGCCGCCAATATCTACCCGAGCAGTCACGTGACCCATCCGCACAGTCACGTGTCACCGCACATAGGC
ACGTATTACGGGTAGACTGTGACGATGCCGGTTGTTGTTTTGACTGATAAAAGTGCCATGGTTTAAGAA
AAACACTCGAATACCGATGGTTAAAAAAAAACGTAACGAACTTACTCCAGCCTGTTTTTAAACGGCATC
TTCAACAACCAGGATTAAGGTAGCCGCCGAGGTACCTTGTGGTTATTTTTAATCGTGAAACTGA

>Pp-clect

CTCTTCCGATCTCCAAACAGTAAGGTCACCGTGTTTGTAACTAGGACAGTATGTTGGCTTGTACTTGTTA
GTTGGTCAGTCAGTGGTTGGTTGTTTGGTAGGAGTAAGTCAGTTCTTGTAGGCCAAGTAAATAAGCAG
TTCACTTGTTTTGTTGGTCGGTCAGTAATTCGTTTGTTGGTCAGTTGGTTGACCTGTAAGTTGGTTGGTT

AGTAAACCGCTGAGGGCGCATCAGACGGTTTGCAGTCGGTCATTTTATAGTTGGCCAGTATGCGGTGT
TCAGTAGATAGCCTCAAAGCATTTTTGTTCGTTGGTTAGTTGCTTGGCTGTAGGTTGATATGTTTGCCAT
TGCAATACCAACCACCCAACAACCATCCAACTGAATAGCTGAACTATACAGCGAATACCAAAAATAAGG
AGGCCGTCTCCCTCTAGAAAGTGAAACATTTTGAGAAAAAAATAATTTGGCTAAAATTACATTGTCATA
TTGACTAAAATATCCTGAAGGACAATTAACAACAGCTGATGTGGCAGCCTGAGTGTTCTATGTAAACAG
GAGGTATCTCTTTATACGGGCCTCAATTATTTTCTTAACTTCCAAATTAGTTCTTATCCTATGAGTCCCCG
TTTTCCTGTTGTGAAGCTGGACAAGGGACTTGGCTCCGAACAAACTGCATAATGCATGATTGACTCATC
CCTGTTGCATATGCAAATAAATGGGCGGGTCGCAAAAGTTTACCGCGCATGGGCACACGAGTTCGCGA
TGCTGTAGACTAGGTTTGATCATTTTTGTTTGGCTTTTATAGTCGACCTGCATTCGAGGTGCAGCTCGCC
TGAGACACAGATCGACACCCAGGTTGAGGAACAGACCCCCAGCCGTTCAAAATGCAGGTTTTCTCCCCT
TGCTTGTTGGTTCTACTGGTCGCCGGCCTGGCCACGGCCCAACGGCTCAACAACTGCCCGACGATGTGG
TCCGGCGCCGGCGAATACTGCTACCAGTTCTTCAAGCAGCCTCTGCCCTGGCAGGAAGCGAGCAACTTC
TGCTCCAAGTTCTCCTCCTGCGCCACGGGAGCGGCGGCCAGCCTGCTGAAGCTCGACTCACCCCAGGCC
GACACGCGGCTGGAGAAATTCCTGAGTCCAAAAGGCAACCCCAGCCCTCCCAACACGTGGATCGGCTT
GGGCGATCCCATGAACACGGGTCAGCTCACCTGGCCTGACGGTACCACCACACAGAGCGTAGGCTACA
ACAACTTCGGGCAGGGAGGCCGGAACGGAAACTGCGCTCTGAAGGACACCCGCACGAACCAGTGGAC
CTTCGCGCCCTGCACGCAAGCGAACTCCTTCGTCTGTCAGATGCAGCGGAGCGCCCCTCTGCAGACCGG
CGGCCCAGGCACCAATTTCCCTTCACGACCTGTCTAAAAGTCGGCTCGGACGAACAATTTCACGAACAA
TTTGTGCAGCGAACAAGAGGAATTCGTACAAAAACTGAACAACCCAATCGGGATCCAAGATGGCCGTA
CGAGAAGTAGCACCAAGTATAGTTCCCTGCCCCAGTCTCACCAACATCCAGCGTGTTTTCATCCAGTATA
GGTCGAGTACCGGCGATGACCATCAAAAAGTATTTGCTCCACTCTGTAAAACTTCTTCTGGTACAAACA
TTCATCGCTATCTTGTGAAGCTTTCTTCAGATGTGAGCAATAATTGAACTACAGAAAAAAATGACAGCA
TTCACTGAACAGTCGGACACGAATTTATCGAATAGTAACGCATTTTATCGAATATGTTATCTCATTTTGT
GTTAGTTTGTGTTAGCACTTCTTTGACAATAATGTTGGCAACGGGATTTTCAAGAAACGAATAAAGGAG
AATCAGTGGTCCATGAAATAAAAAAAAAGATCGGAAGAGCAC
